# Thyroid follicular cell-derived GDF15 attenuates inflammation and lipid dysregulation in Hashimoto's thyroiditis

**DOI:** 10.3389/fmed.2026.1854594

**Published:** 2026-07-22

**Authors:** Ruili Yin, Rongxin Sun, Boshen Gong, Zhongyan Shan, Dong Zhao

**Affiliations:** 1 Center for Endocrine Metabolism and Immune Diseases, Beijing Luhe Hospital, Capital Medical University, Beijing, China; 2 Department of Endocrinology and Metabolism, NHC Key Laboratory of Diagnosis and Treatment of Thyroid Diseases, The First Hospital of China Medical University, Shenyang, China

**Keywords:** GDF15, Hashimoto's thyroiditis, inflammation, lipid metabolism, thyroid follicular epithelial cells

## Abstract

**Introduction:**

Hashimoto's thyroiditis (HT) is a prevalent autoimmune thyroid disorder, typically triggers localized thyroid inflammation and systemic dyslipidemia, and progression to hypothyroidism. While growth differentiation factor 15 (GDF15) is recognized for its role in lipid metabolism and inflammatory diseases, its specific involvement in HT remains elusive.

**Methods:**

We conducted plasma metabolomic profiling in HT patients and matched healthy controls to identify altered metabolic pathways. Correlation analysis was performed between serum GDF15 and clinical indicators. In vitro thyroid follicular cell models and NaI-induced in vivo thyroid inflammatory models were applied with GDF15 overexpression, knockdown, recombinant protein and siRNA treatment to explore the functional role of GDF15.

**Results:**

Plasma metabolomic profiling in HT patients revealed that differentially expressed metabolites are predominantly enriched in lipid metabolism pathways. GDF15 expression was increased by 1.98-fold in HT serum compared to controls (*p* < 0.001), and serum GDF15 levels were negatively correlated with thyroid-stimulating hormone (TSH, *R* = −0.4943, *p* = 0.0040), aspartate aminotransferase (AST, *R* = −0.4506, *p* = 0.0238) and ultrasound attenuation parameter (UAP, *R* = −0.4894, *p* = 0.0071) in HT patients. Functional assays demonstrated that GDF15 overexpression tends to attenuate NaI-induced thyroid and systemic inflammation, relatively improving lipid profiles and reduces hepatic lipid deposition; however, GDF15 knockdown appeared to exacerbate these pathologies. Furthermore, recombinant GDF15 protein mitigates NaI-induced inflammatory responses in thyroid follicular cells, whereas *GDF15* siRNA intensifies the inflammation.

**Discussion:**

Given the limitations of the NaI-induced model and the lack of thyroid-specific genetic manipulation, these findings suggest a significant association between GDF15 and HT, though further research is required to establish definitive causality.

## 1 Introduction

Autoimmune thyroiditis (AIT) encompasses a spectrum of inflammatory disorders driven by autoimmune etiologies, characterized by an immune-mediated assault on thyroid tissue that culminates in thyroid dysfunction (1). The clinical spectrum of autoimmune thyroid disorders encompasses two polar phenotypes: Graves' disease, associated with hyperthyroidism, and Hashimoto's thyroiditis (HT), which leads to hypothyroidism. HT, the most prevalent form, initially presents with elevated titers of anti-thyroperoxidase antibody (TPOAb) and anti-thyroglobulin antibody (TgAb), accompanied by chronic inflammation and characteristic ultrasonographic features; it subsequently progresses to hypothyroidism, dyslipidemia, and impaired bone metabolism (2–5).

Chronic inflammation serves as the cornerstone of the pathogenesis and clinical progression of HT (6). At the cellular level, breakdown of immune tolerance results in extensive intrathyroidal infiltration of CD4^+^ Th1 cells, B cells, and macrophages (7–9). These infiltrating cells orchestrate a “cytokine storm” within the gland, releasing pro-inflammatory mediators such as interferon-gamma (IFN-γ), tumor necrosis factor-alpha (TNF-α), and interleukin-6 (IL-6) (9, 10). These cytokines not only trigger thyrocyte apoptosis via death receptor pathways but also exacerbate tissue damage through the induction of oxidative stress, ultimately culminating in progressive fibrosis and glandular atrophy (11). This sustained inflammatory state is intimately associated with multisystemic clinical manifestations in HT patients, including insulin resistance, obesity, fatigue, and psychological distress (12–15). Consequently, chronic inflammation acts not only as the primary driver of thyroid hypofunction but also as the underlying pathological basis for the multisystemic clinical manifestations of the disease.

Growth and Differentiation Factor 15 (GDF15) is a pivotal mediator linking systemic inflammation to metabolic regulation (16, 17). Beyond its established role in appetite suppression via the brainstem-localized GDNF family receptor alpha like (GFRAL) receptor, GDF15 plays a role in body weight regulation and metabolic health (18, 19). Luan et al. (20) reported that GDF15 exerts a protective effect during acute inflammation by activating hepatic sympathetic nerve signaling and promoting triglyceride synthesis, thereby meeting cardiac metabolic demands, preventing heart damage, and averting heart failure. However, the expression changes and functional role of GDF15 in HT remain elusive, and systematic investigation of the immune-metabolic effects of GDF15 modulation is lacking.

Based on our previous metabolomic findings, we identified that differential metabolites were predominantly enriched in lipid metabolism signaling pathways (21). Subsequent clinical analysis of HT patients revealed significantly elevated GDF15 levels in both serum and thyroid tissues. To further elucidate its functional role, we utilized NOD mouse models subjected to GDF15 overexpression and knockdown, assessing thyroid inflammation alongside hepatic and systemic lipid profiles. Our results demonstrate that GDF15, secreted by thyroid follicular cells, improves thyroid function and systemic lipid metabolism in HT by suppressing inflammation and reducing hepatic lipid accumulation.

## 2 Material and methods

### 2.1 Patients samples

A total of 30 patients with HT and 26 healthy controls were consecutively recruited from Beijing Luhe Hospital between January 2021 and December 2023. All participants were enrolled via consecutive recruitment rather than random sampling. Healthy controls were individually matched with HT patients in terms of age and body mass index (BMI) at a ratio close to 1:1. This study was approved by the Ethics Committee of Beijing Luhe Hospital (ethics approval number: 2021-LHKY-090-04). Basic biochemical and clinical parameters of all serum sample participants are summarized in Table 1.

**Table 1:** General clinical and laboratory parameters in all participants.

Variables	Control (*n* = 26)	HT (*n* = 30)	*p* value
Sex (M/F)	14/12	7/23	**0.019** ^*^
Age (y)	47.27 ± 9.89	43.42 ± 12.44	0.212
BMI (Kg/m^2^)	24.96 ± 4.01	23.87 ± 5.84	0.426
SBP (mmHg)	124.03 ± 19.01	128.47 ± 17.64	0.353
DBP (mmHg)	80.13 ± 12.87	82.30 ± 10.75	0.482
LSM (mmol/L)	7.18 ± 3.16	6.65 ± 2.33	0.474
UAP (mmol/L)	262.77 ± 34.60	256.53 ±27.01	0.293
ALT (U/L)	22.04 ± 13.91	24.93 ± 12.67	0.434
AST (U/L)	22.96 ± 10.78	22.83 ± 8.22	0.960
T3 (ng/mL)	1.14 ± 0.23	1.11 ± 0.18	0.593
T4 (μg/dL)	7.57 ± 0.97	7.97 ± 1.45	0.223
FT3 (pg/mL)	3.04 ± 0.47	3.14 ± 0.48	0.442
FT4 (ng/dL)	1.30 ± 0.14	1.29 ± 0.19	0.920
TSH (μIU/mL)	1.56 ± 0.72	2.16 ± 0.98	0.012
TgAb (U/mL)	18.42 ± 1.49	2.87 ± 0.90	< 0.0001^*^
TPOAb (U/mL)	2.58 ± 1.05	2.87 ± 0.90	< 0.0001^*^
GDF15 (pg/mL)	100.91 ± 85.03	200.30 ± 105.98	< 0.0001^*^

^*^p < 0.05 was considered significant. T3, triiodothyronine; T4, tetraiodothyronine; FT3, free triiodothyronine; FT4, free tetraiodothyronine; TSH, thyroid-stimulating hormone; TgAb, thyroglobulin antibody; TPOAb, thyroid peroxidase antibody; ALT, alanine aminotransferase; AST, aspartate transaminase; After adjustment for gender and TSH level using analysis of covariance (ANCOVA), the difference in GDF15 levels between the two groups remained statistically significant (*F* = 13.184, *p* = 0.002). All thyroid function and autoantibody indicators were interpreted based on the manufacturer's reference ranges as follows: FT3 (2.02–4.43 pg/mL), FT4 (0.93–1.71 ng/dL), TSH (0.27–4.2 μIU/mL), TgAb (< 115 U/mL) and TPOAb (< 34 U/mL).

Informed consent was obtained from healthy control individuals and HT patients. The thyroid tissue sections used in this study were obtained from samples retained during fine-needle aspiration procedures in cases of nodular goiter, categorized into Control (*n* = 4) and HT (*n* = 4) groups. The clinical characteristics of all participants are detailed in Table 2. Eligible HT patients were aged 18–65 years and euthyroid [normal free triiodothyronine (FT3), free thyroxine (FT4), and thyroid-stimulating hormone (TSH)] without history of hormone therapy. Reference values provided by the manufacturer were: FT3 (2.02–4.43 pg/mL), FT4 (0.93–1.71 ng/dL), TSH (0.27–4.2 μIU/mL), TgAb (< 115 U/mL) and TPOAb (< 34 U/mL).

**Table 2:** General clinical and laboratory parameters in HT and control participants.

Index	Control	Control	Control	Control	HT	HT	HT	HT
Sex	Female	Female	Female	Male	Female	Female	Female	Male
Age (year)	70	58	55	39	60	62	54	71
T3 (ng/mL)	0.77	0.89	0.9	1.08	0.94	1.16	1.27	1.25
T4 (μg/dL)	4.96	9.3	6.16	5.45	5.24	7.07	9.11	9.58
FT3 (pg/mL)	2.94	2.73	2.95	3.73	2.93	3.02	2.83	2.95
FT4 (ng/dL)	1.02	1.22	1.14	1.16	1.08	1.18	1.13	1.3
TSH (μIU/mL)	2.93	1.01	1.47	0.83	2	2.12	2.65	0.82
TgAb (U/mL)	10.8	0.33	10.7	13.4	503	414	302	13.2
TPOAb (U/mL)	< 9.00	< 28	< 9.00	< 9.00	9.5	74.5	157	207
ALT (U/L)	14	10	21	50	33	18	18	18
AST (U/L)	17	13	16	29	33	16	17	18
FBG (mmol/L)	6.21	6.8	5.31	5.92	4.98	5.35	8.42	5.27
TG (mmol/L)	0.59	1.26	1.02	0.79	0.85	1.64	1.19	2.18
CHO (mmol/L)	3.87	4.27	3.9	4.69	4.42	7.56	5.29	5.15
HDL-C (mmol/L)	1.66	1.11	1.5	1.36	1.27	1.5	1.68	1.23
LDL-C (mmol/L)	2.08	2.91	3.18	2.86	2.99	5.01	3.14	3.26

^*^p < 0.05 was considered significant. T3, triiodothyronine; T4, tetraiodothyronine; FT3, free triiodothyronine; FT4, free tetraiodothyronine; TSH, thyroid-stimulating hormone; TgAb, thyroglobulin antibody; TPOAb, thyroid peroxidase antibody; ALT, alanine aminotransferase; AST, aspartate transaminase; FBG, fasting blood glucose; TG, triglyceride; CHO, cholesterol; HDL-C, high-density lipoprotein cholesterol; LDL-C, low-density lipoprotein cholesterol.

Data on liver stiffness and steatosis were obtained from participants at the Department of Endocrinology, Capital Medical University, between March 2025 and September 2025. All examinations were conducted in the Ultrasound Room of the Center for Endocrine Metabolism and Immune Diseases. Liver stiffness measurement (LSM), ultrasound attenuation parameter (UAP), and liver iron detection (LID) were assessed using a quantitative shear wave ultrasound elastography system (Plus8500, Wuxi, China) equipped with abdominal ultrasound probes (YD-C1, Wuxi, China). This technology utilizes vibration-controlled transient elastography to provide non-invasive quantification of hepatic fibrosis and steatosis.

Comprehensive exclusion criteria were applied as follows: (1) participants with diabetes mellitus, hypertension, liver diseases, malignant tumors, acute infection or chronic inflammatory disorders; (2) pregnant or lactating women; (3) individuals who had taken levothyroxine, statins, glucocorticoids, antibiotics, probiotics or iodine-containing drugs within the preceding 3 months; (4) patients with chronic diarrhea. Serum levels of TSH, TPOAb and TgAb were detected using a Cobas 601 analyzer (Roche Diagnostics). Fresh morning fecal samples were collected using sterile instruments, immediately snap-frozen in liquid nitrogen and stored at −80 °C until further analysis.

### 2.2 Animals and treatment

NOD.H-2h4 mice, which spontaneously develop autoimmune thyroiditis, were purchased from the Jackson Laboratory (Bar Harbor, ME, USA). All animal procedures were conducted in strict accordance with the established guidelines for the care and use of laboratory animals. Female NOD.H-2h4 mice (5–6 weeks old) were utilized in this study. The mice were randomly assigned to receive drinking water supplemented with sodium iodide (NaI) at concentrations of 10 mg/L or 50 mg/L for a duration of 12 or 16 weeks (21). Mice were randomly assigned to each group using a random number table. Sample size (*n* = 6 per group) was determined based on a pilot study showing a 1.5-fold difference in GDF15 expression with 80% power at α = 0.05. All histological assessments were performed by a blinded investigator. The control group received regular drinking water throughout the study. The successful establishment of the HT model was validated through histological examination of the thyroid gland via Hematoxylin and Eosin (H&E) staining and the level of serum TgAb concentrations.

### 2.3 Overexpression and knockdown of GDF15 in mice

For the overexpression and knockdown of GDF15, we constructed adeno-associated virus serotype 8 (AAV8) vectors under the control of the cytomegalovirus (CMV) promoter, namely AAV8-p*Gdf15* and AAV8-sh*Gdf15* (Supplementary Figure 1). Given the technical difficulty of in-situ injection into the thyroid gland, we delivered a dose of 1 × 10^12^ vg/mL of the corresponding AAV8 vectors via intravenous injection to each mouse to achieve GDF15 overexpression or knockdown„ and each mouse received an injection volume of 100 μL. Eight weeks after NaI administration, the mice were subjected to the above intravenous injection. Serum GDF15 levels were subsequently measured to verify the efficiency of GDF15 overexpression and knockdown. Serum triglyceride (TG, NJJCBIO, A110-1-1, China), total cholesterol (TC, NJJCBIO, A111-1-1, China), alanine transaminase (ALT, Beyotime, P2711M, China) and aspartate aminotransferase (AST, Beyotime, P2715M, China) levels were determined according to standard protocols. Liver samples were collected for the determination of TG (Applygen, E1013, China) contents.

### 2.4 Cell culture and treatment

The normal human normal thyroid follicular epithelial cell line Nthy-ori 3-1 was purchased from the American Type Culture Collection (ATCC, USA) and cultured in RPMI DMEM 1640 (Gibco, USA) with 10% FBS (ExCell, China) and 1% penicillin-streptomycin (Gibco, USA). The mouse leukemic monocyte-macrophage cell line RAW264.7 was purchased from the American Type Culture Collection (ATCC, USA) and cultured in DMEM (Gibco, USA) with 10% FBS (ExCell, China) and 1% penicillin-streptomycin (Gibco, USA). The human hepatocyte cell line HepG2 was cultured in DMEM (Gibco, USA) and mouse normal hepatocyte cell line AML12 was cultured in DMEM/F12 (Gibco, USA) with 10% FBS (ExCell, China) and 1% penicillin-streptomycin (Gibco, USA). All cells were cultured at 37 °C in an incubator with 5% CO_2_ and 95% air humidity. Nthy-ori 3-1 cells were treated with 10 or 50 mM NaI and treated with reconstitution human GDF15 (MedChemExpress, HY-P75171, USA) or transfected with small interfering RNA (siRNA) targeting GDF15 (si*GDF15*), which conducted in Oligobio (< city>Beijing < /city>, China) for 24 h before analysis. Lipopolysaccharide (LPS) was purchased from Sigma (L4391, USA), and for further assays, cells were seeded at a density of 20 × 10^4^ cells/well in six-well plates and treated with LPS (200 ng) and IFN-γ (HY-P7071, MCE, USA) or 20 ng/mL interleukin-4 (IL-4, HY-P7080, MCE, USA) for 24 h, with or without reconstitution mouse GDF15 (MedChemExpress, HY-P77945A, USA).

The Nthy-ori 3-1 cells were transfected with si*GDF15* and treated with NaI for 24 h. The cell culture supernatant was collected, centrifuged at 13,000 g for 20 min at 4 °C, and collected as conditioned medium. GDF15 protein level in this conditioned medium was measured by ELISA. This conditioned medium was added to cultured RAW264.7 or HepG2 or AML12 cells at a 1/3 volume of the total culture volume, and the cells were incubated for 24 h before proceeding with further analyses.

### 2.5 Enzyme-linked immunosorbent assay (ELISA)

Human serum GDF15 levels were determined by a human GDF15 ELISA kit (CUSABIO, CSB-E12009h, China) according to the manufacturer's instructions. For mice, serum GDF15 levels were measured using a mouse GDF15 ELISA kit (Mlbio, YJ665241, China), which exhibited an inter-assay CV of 5.0%−6.3% and an intra-assay CV of 2.6%−7.3%. Serum FT3 (Mlbio, ml300954-J, China), TNF-α (Mlbio, YJ0020953, China) and Interleukin-1β (IL-1β, Mlbio, YJ098416, China) levels were analyzed using a mouse ELISA kit according to the manufacturer's introductions.

### 2.6 Histopathological staining

Thyroid and liver tissues from mice were initially fixed with paraformaldehyde, subsequently embedded in paraffin, and finally sectioned into paraffin sections. Paraffin sections were subjected to HE staining. For histological analysis of hepatic lipids, liver samples were snap-frozen in liquid nitrogen, embedded in OCT medium, and processed into cryosections for subsequent Oil Red O staining.

### 2.7 Immunohistochemical staining (IHC)

After deparaffinization, thyroid paraffin sections were incubated with primary antibodies against GDF15 (Abcam, ab39999, USA), Arginase-1 (ARG1, Novus Biologicals, NBP1-32731, USA), and F4/80 (Proteintech, 28463-1-AP, USA), followed by incubation with a horseradish peroxidase-conjugated secondary antibody (MXB Biotechnologies, KIT-9720, China). After dehydration and mounting, slides were examined microscopically. Positive staining was quantified with ImageJ software.

### 2.8 Western blot

Cells were homogenized in ice-cold RIPA buffer containing protease and phosphatase inhibitors. Following a 30-min incubation on ice, the samples were centrifuged at 4 °C to clarify the lysates, and the protein-containing supernatants were collected. Following visualization, the optical density of the target bands was determined using ImageJ software.

### 2.9 RNA isolation and real-time polymerase chain reaction (RT-qPCR)

Total RNA was isolated from thyroid tissues, other selected tissues, and cultured cells using TRIzol reagent (Thermo Fisher, USA). The extracted RNA was then reverse transcribed into complementary DNA (cDNA) using HiScript^®^ All-in-one RT SuperMix (Vazyme, R333-01, China). qPCR was subsequently conducted on a Bio-Rad CFX96 Real-Time PCR Detection System with ChamQ Universal SYBR qPCR Master Mix (Vazyme, Q711-03, China). All primer sequences used in this study are listed in Table 3. The mRNA expression levels of target genes were normalized to that of β-actin as an internal control.

**Table 3:** The primers sequences in the study.

Primer	Species	Forward primer (5^′^-3')	Reverse primer (5'-3')
*Gdf15*	mice	TGGGTTCTAGCCAGCAGAGT	ACCACCAGAGACCGTTATGC
*Tnf-α*	mice	CCCTCACACTCACAAACCAC	ACAAGGTACAACCCATCGGC
*Il-1b*	mice	TGCCACCTTTTGACAGTGATG	ATGTGCTGCTGCGAGATTTG
*Il-6*	mice	ACAAAGCCAGAGTCCTTCAGAG	TGTGACTCCAGCTTATCTCTTGG
*Nlrp3*	mice	CAAGGCTGCTATCTGGAGGAA	TGCAACGGACACTCGTCATC
*iNos*	mice	GTTCTCAGCCCAACAATACAAGA	GTGGACGGGTCGATGTCAC
*Arg1*	mice	CTCCAAGCCAAAGTCCTTAGAG	AGGAGCTGTCATTAGGGACATC
*Cd206*	mice	CTCTGTTCAGCTATTGGACGC	CGGAATTTCTGGGATTCAGCTTC
*NLRP3*	human	ATGTGCCAGAGGAGCTGAGT	TGATCCACTGTTGCTTCTGC
*ASC*	human	TTCAACCGAGTGGGTTATTTTGT	TACTGGGCATCTGGGGTGTAG
*GDF15*	human	ACTTCTGGAGACATCGCAAAC	GGTAGACAACAGCCGCATC
*NLRP1*	human	CTGGGTCTGGTTCAGGGATG	GCTGGTGTTCCTTCCTGGTTTC
*NLRC4*	human	AGGCCTCACTGAAACGGAA	AAACTACTCTTCATTCTGGCTGA
*TNF-α*	human	GCCCATGTTGTAGCAAACCCT	CCTTGAAGAGGACCTGGGAG
*IL-1b*	human	TTCGAGGCACAAGGCACAA	TGGCTGCTTCAGACACTTGAG
*IL-6*	human	TGAACTCCTTCTCCACAAGCG	CCGTCGAGGATGTACCGAAT
*β-actin*	human/mice	TCATGAAGTGTGACGTGGACATC	CAGGAGGAGCAATGATCTTGATCT

### 2.10 Oil Red O staining of cultured cells

The cells were fixed with 4% paraformaldehyde for 30 min at room temperature, stained with Oil Red O Stain Kit (Solarbio, G1262, China) for 20 min. Images were captured using microscopy, and lipid area percent was quantified.

### 2.11 Plasmid and siRNA construction

The human *GDF15* siRNA were constructed using Oligobio (Beijing, China). The siRNAs were transfected into cells using Lipofectamine 2000 (11668019, Thermo Fisher, USA) according to the manufacturer's instructions.

### 2.12 Statistical analysis

All statistical analyses were performed using IBM SPSS Statistics 25.0 and GraphPad Prism 10.1.2. Continuous variables were expressed as mean ± standard deviation (SD), and categorical variables were presented as frequencies and percentages. For between-group comparisons of baseline characteristics, independent-samples *t*-test was used for normally distributed continuous variables, and the chi-square test was applied for categorical variables. Pearson correlation analysis was used to assess correlations. For two-group comparisons, the independent-samples *t*-test was adopted for normally distributed data, while the Mann–Whitney U test was used for non-normally distributed data. One-way analysis of variance (ANOVA) and Kruskal–Wallis H test was performed for multiple-group comparisons, respectively. Analysis of covariance (ANCOVA) was used to compare the levels of continuous indicators such as GDF15 between the two groups. Sex and TSH were included as covariates to adjust for potential confounding factors. The homogeneity of regression slopes was verified prior to ANCOVA. Statistical significance was set at *p* < 0.05.

## 3 Results

### 3.1 Elevated serum GDF15 correlates with thyroid function in HT patients

**Figure 1:**
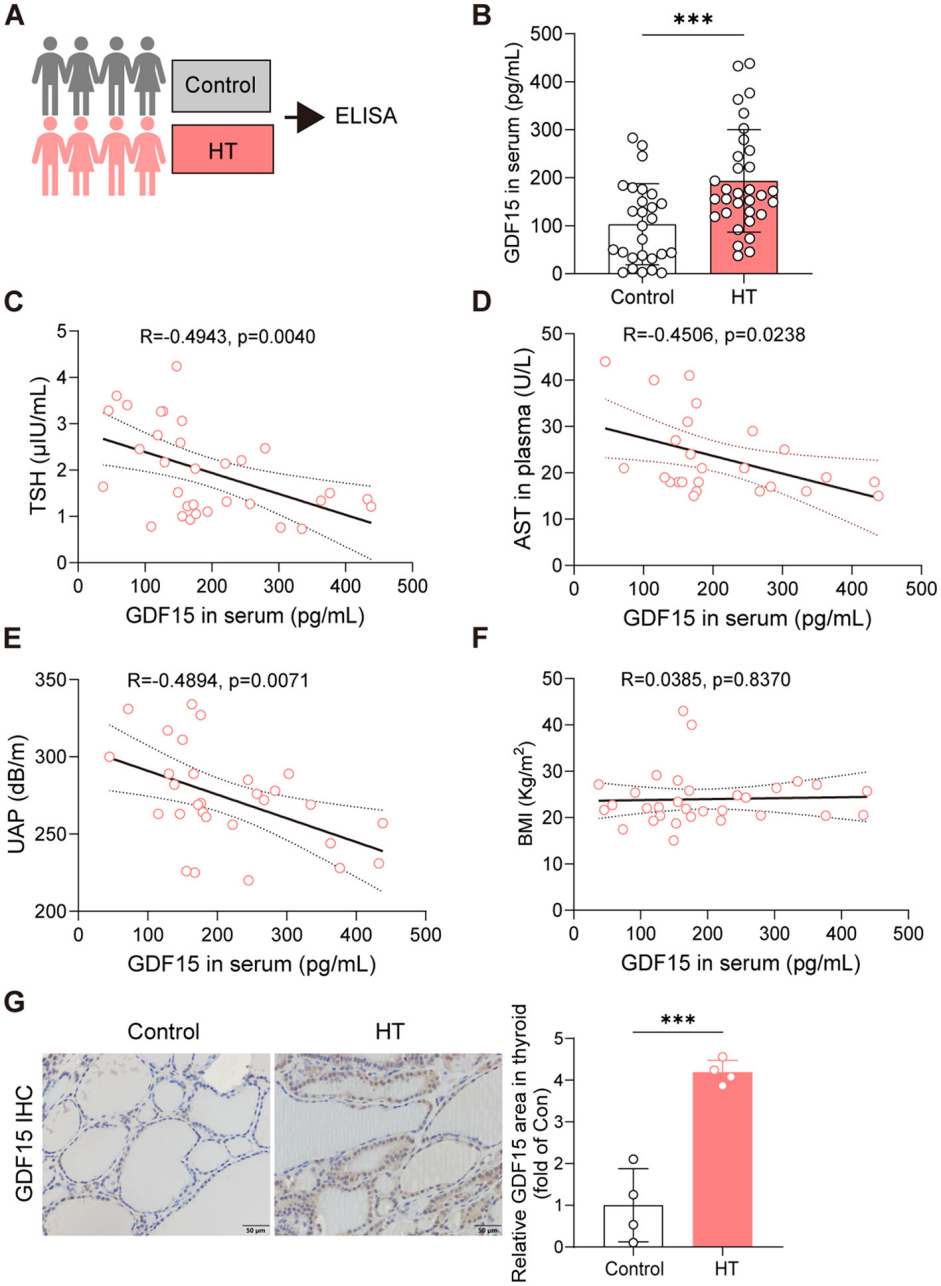
GDF15 levels are upregulated in serum and thyroid of patients of HT. **(A)** Schematic overview of human study. **(B)** Serum GDF15 levels in HT (*n* = 30) and Control participants (*n* = 26). **(C–F)** Correlation analysis between serum GDF15 levels and TSH, AST, UAP and BMI (*n* = 30). **(G)** Representative images of GDF15 IHC staining in patients with HT and Control (left panel). Statistical plot of positive area in the liver (right panel, *n* = 4). Scale bar: 50 μm. ***p < 0.001.

To investigate the mechanisms underlying HT-induced metabolic dysregulation in patients, we initially conducted metabolomic profiling (21), which revealed that differentially abundant metabolites were primarily enriched in lipid metabolism pathways (Supplementary Figures 2A–C). Subsequently, we enrolled 30 treatment-naive HT patients and 26 age/BMI -matched healthy controls. All HT patients were diagnosed based on clinical guidelines, and those on levothyroxine or other thyroid-related medications were excluded. Serum GDF15 were 1.98-fold higher in HT patients (*p* < 0.001; Figures 1A, B), which was negatively correlated with TSH (*R* = −0.4943, *p* = 0.0040), AST (*R* = −0.4506, *p* = 0.0238), and UAP (*R* = −0.4894, *p* = 0.0071; Figures 1C–E), but not correlated with BMI (Figure 1F). After adjusting for gender and TSH level using analysis of covariance (ANCOVA), the difference in GDF15 levels between the two groups remained statistically significant (*F* = 11.254, *p* = 0.002). Subsequently, we found that GDF15 is predominantly expressed in thyroid follicular cells, and its expression is significantly increased in thyroid tissues from patients with HT (Figure 1G). The above findings suggest that GDF15 may be involved in thyroid tissue dysfunction in patients with HT.

### 3.2 Thyroid-derived GDF15 expression is elevated in HT mouse model

**Figure 2:**
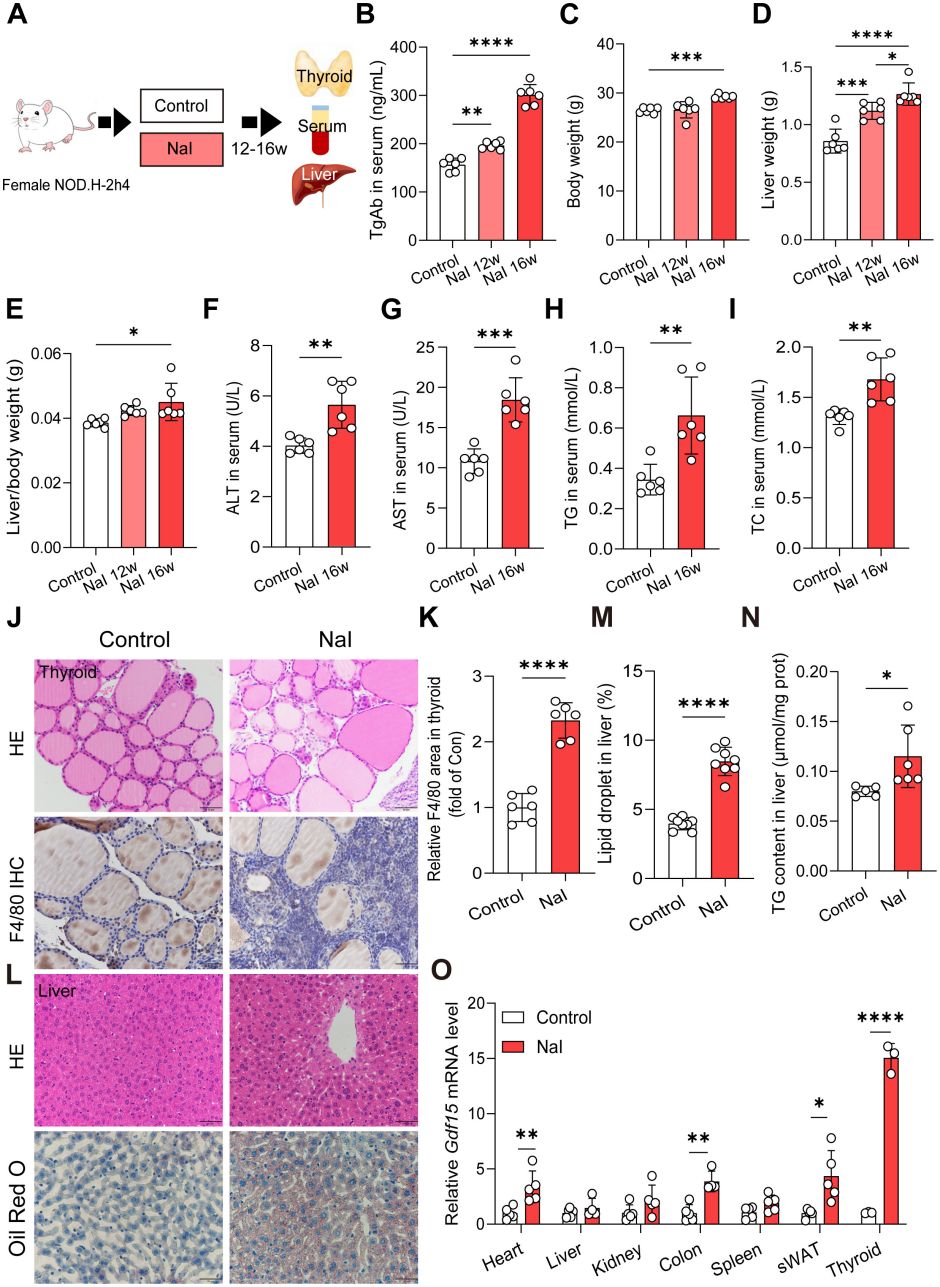
Thyroid-derived GDF15 expression is elevated in HT mouse model. **(A)** Schematic overview of NaI-induced HC mouse model. **(B)** Serum TgAb levels were measured in mice 12–16 weeks after NaI treatment (*n* = 6). **(C–E)** Body weight **(C)**, liver weight **(D)**, liver/body weight ratio **(E)** were determined in mice 12–16 weeks after NaI treatment (*n* = 6). **(F–I)** Serum ALT **(F)**, AST **(G)**, TG **(H)** and TC **(I)** levels were measured in mice 16 weeks after NaI treatment (*n* = 6). **(J)** Representative images of HE (upper panel) and F4/80 IHC staining (lower panel) of thyroid tissues from 16 weeks NaI treatment. Scale bar: 50 μm. **(K)** Statistical data of the positive area of F4/80 in the thyroid (*n* = 6). Scale bar: 50 μm. **(L)** Representative images of HE (upper panel) and Oil Red O staining (lower panel) of liver tissues from 16 weeks NaI treatment. Scale bar: 50 μm. **(M)** Quantification of lipid droplet area using Oil Red O staining (*n* = 6). **(N)** Hepatic TG content in 16 weeks NaI treated mice (*n* = 6). **(O)** Changes in mRNA levels of *Gdf15* in heart, liver, kidney, colon, spleen, sWAT and thyroid (*n* = 3–6). sWAT, subcutaneous white adipose tissue; GDF15, growth differentiation factor 15. *p < 0.05, **p < 0.01, ***p < 0.001, ****p < 0.0001.

Given that thyroid follicular cells are the primary cell type in the thyroid and are directly exposed to autoimmune attack, we hypothesized that locally derived GDF15 may exert paracrine protective effects distinct from its systemic actions. We established a mouse model of NaI-induced HT mouse model (Figure 2A). With the extension of NaI treatment duration, serum TgAb levels gradually increased (Figure 2B), while body weight, liver weight, and liver-to-body weight ratio showed significant increase at 16 weeks (Figures 2C–E). To clarify the role of GDF15 in lipid metabolism during HT progression, subsequent experiments were conducted using an animal model treated with NaI for 16 weeks. Serum ALT, AST, TG, TC levels were elevated in NaI-treated mice (Figures 2F–I). Thyroid inflammation was assessed by HE and F4/80 IHC staining (Figures 2J–K). Hepatic steatosis was confirmed by HE and Oil Red O staining, which revealed increased lipid accumulation. This finding was further corroborated by a quantitative increase in hepatic triglyceride content (Figures 2L–N). These results indicate that the NaI-induced mouse model of Hashimoto's thyroiditis exhibits increased thyroid inflammation and concomitant abnormal hepatic lipid metabolism. Although GDF15 is known to be widely expressed, analysis of mRNA levels across tissues revealed the thyroid gland as the primary source of GDF15 in this model (Figure 2O). The precise role of thyroid-derived GDF15 in HT remains unclear.

### 3.3 Observations of inflammatory and hepatic lipid phenotypes in NaI-induced HT mice with systemic GDF15 overexpression

**Figure 3:**
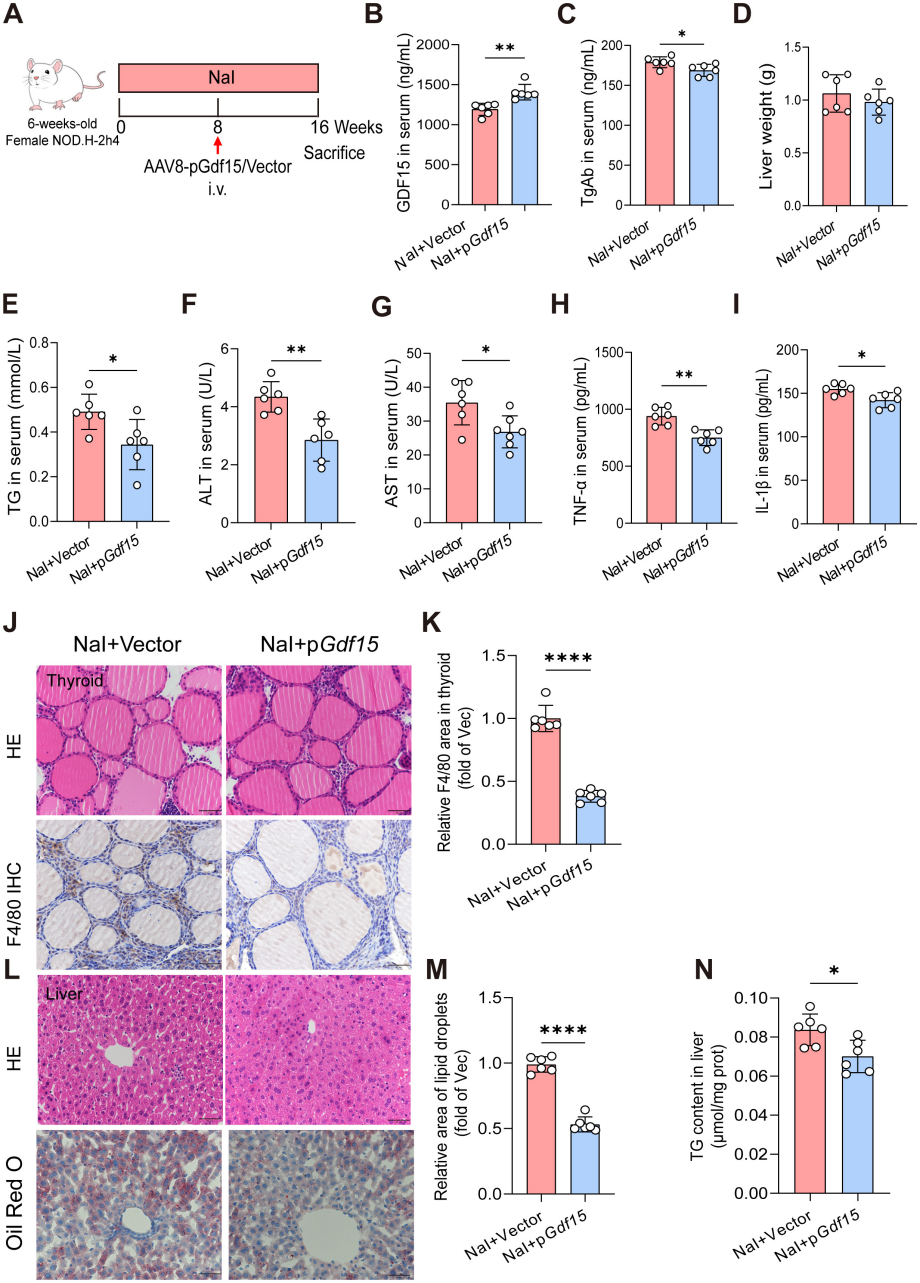
Observations of inflammatory and hepatic lipid phenotypes in NaI-induced HT mice with systemic GDF15 overexpression. **(A)** Flow chart illustrates the injection of AAV8-p*Gdf15* or AAV8-Vector and the treatment protocol in the NaI-induced HT mouse model. **(B, C)** Serum GDF15 **(B)** and TgAb **(C)** levels were measured in mice 12–16 weeks after NaI treatment (*n* = 6). **(D)** Liver weight was determined in mice 12–16 weeks after NaI treatment (*n* = 6). **(E–I)** Serum TG **(E)**, ALT **(F)**, AST **(G)**, TNF-α **(H)** and IL-1β **(I)** levels were measured in mice 16 weeks after NaI treatment (*n* = 6). **(J)** Representative images of HE (upper panel) and F4/80 IHC staining (lower panel) of thyroid tissues from 16 weeks NaI treatment. Scale bar: 50 μm. **(K)** Statistical data of the positive area of F4/80 in the thyroid (*n* = 6). **(L)** Representative images of HE (upper panel) and Oil Red O staining (lower panel) of liver tissues from 16 weeks NaI treatment. Scale bar: 50 μm. **(M)** Quantification of lipid droplet area using Oil Red O staining (*n* = 6). **(N)** Hepatic TG content in 16 weeks NaI treated mice (*n* = 6). *p < 0.05, **p < 0.01, ***p < 0.001, ****p < 0.0001.

To elucidate the regulatory role of GDF15 in HT, we constructed AAV8-p*Gdf15* or AAV8-Vector. After being treated with NaI for 8 weeks, then injected with AAV8-p*Gdf15* or AAV8-Vector for 8 weeks in mice (Figure 3A). Circulating GDF15 concentrations were significantly elevated in the AAV8-p*Gdf15* group (Figure 3B), which was accompanied by reduced NaI-triggered TgAb levels (Figure 3C). No significant differences were observed in fasting body weight, liver weight, liver and body weight ratio, serum TC and FT3 levels (Supplemetary Figures 3A–D; Figure 3D). Notably, mice with elevated systemic GDF15 exhibited reduced serum TG, ALT, AST, TNF-α and IL-1β levels (Figures 3E–I). Histological analysis (HE and F4/80 IHC) in thyroids showed that GDF15 overexpression significantly decreased thyroid inflammation (Figures 3J, K). Liver HE and Oil Red O staining showed that GDF15 overexpression decreased liver lipid deposition, which was further confirmed by a quantitative assay of hepatic TG content (Figures 3L–N). These results indicate that systemic GDF15 overexpression is associated with milder thyroid inflammation and reduced hepatic lipid storage in the NaI-induced HT mouse model.

### 3.4 Observations of inflammatory and hepatic lipid phenotypes in NaI-induced HT mice with systemic GDF15 knockdown

**Figure 4:**
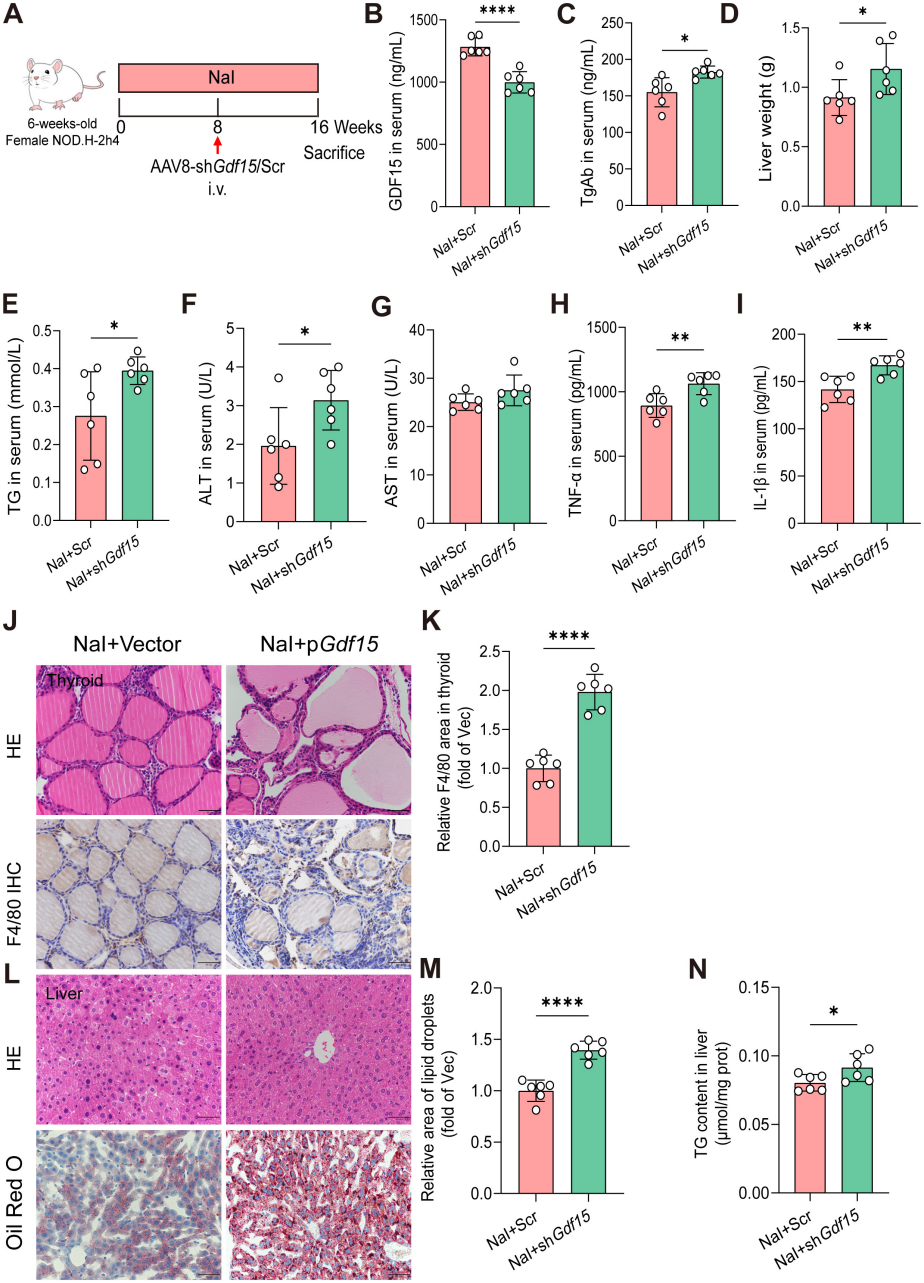
Observations of inflammatory and hepatic lipid phenotypes in NaI-induced HT mice with systemic GDF15 knockdown. **(A)** Flow chart illustrates the injection of AAV8-p*Gdf15* or AAV8-Vector and the treatment protocol in the NaI-induced HT mouse model. **(B, C)** Serum GDF15 **(B)** and TgAb **(C)** were measured in mice 12–16 weeks after NaI treatment (*n* = 6). **(D)** Liver weight was determined in mice 12–16 weeks after NaI treatment (*n* = 6). **(E–I)** Serum TG **(E)**, ALT **(F)**, AST **(G)**, TNF-α **(H)** and IL-1β **(I)** levels were measured in mice 16 weeks after NaI treatment (*n* = 6). **(J)** Representative images of HE (upper panel) and F4/80 IHC staining (lower panel) of thyroid tissues from 16 weeks NaI treatment. Scale bar: 50 μm. **(K)** Statistical data of the positive area of F4/80 in the thyroid (*n* = 6). **(L)** Representative images of HE (upper panel) and Oil Red O staining (lower panel) of liver tissues from 16 weeks NaI treatment. Scale bar: 50 μm. **(M)** Quantification of lipid droplet area using Oil Red O staining (*n* = 6). **(N)** Hepatic TG content in 16 weeks NaI treated mice (*n* = 6). *p < 0.05, **p < 0.01, ***p < 0.001, ****p < 0.0001.

To further examine the association between GDF15 abundance and HT-related pathological manifestations, we utilized AAV8-shGdf15 to achieve systemic GDF15 silencing in vivo. After 8 weeks of NaI treatment, mice received AAV8-sh*Gdf15* or AAV8-Scr for another 8 weeks (Figure 4A). Systemic GDF15 knockdown resulted in decreased circulating GDF15 levels (Figure 4B) and augmented NaI-induced TgAb secretion (Figure 4C). While fasting body weight was slightly increased in the AAV8-sh*Gdf15* group, the liver-to-body weight ratio and FT3 levels remained comparable between groups (Supplementary Figures 3E–G). Additionally, serum TC, liver weight, TG, ALT, TNF-α, and IL-1β levels were elevated in the AAV8-sh*Gdf15* group, but AST levels were not (Supplementary Figure 3H; Figures 4D–I). Histological analysis (HE and F4/80 IHC) showed that GDF15 knockdown worsened thyroid inflammation (Figures 4J, K). Liver HE and Oil Red O staining showed that GDF15 knockdown increased liver lipid deposition, as confirmed by TG quantification (Figures 4L–N). These observations suggest that reduced systemic GDF15 levels correlate with aggravated thyroid inflammation and exacerbated hepatic lipid deposition. Importantly, due to the lack of thyroid follicular cell-specific GDF15 genetic models, these phenotypes should be interpreted as associations with systemic GDF15 levels rather than definitive evidence of thyroid-derived GDF15 function.

### 3.5 Thyroid follicular epithelial cells-derived GDF15 may attenuate NaI-induced inflammation and hepatic lipid accumulation

**Figure 5:**
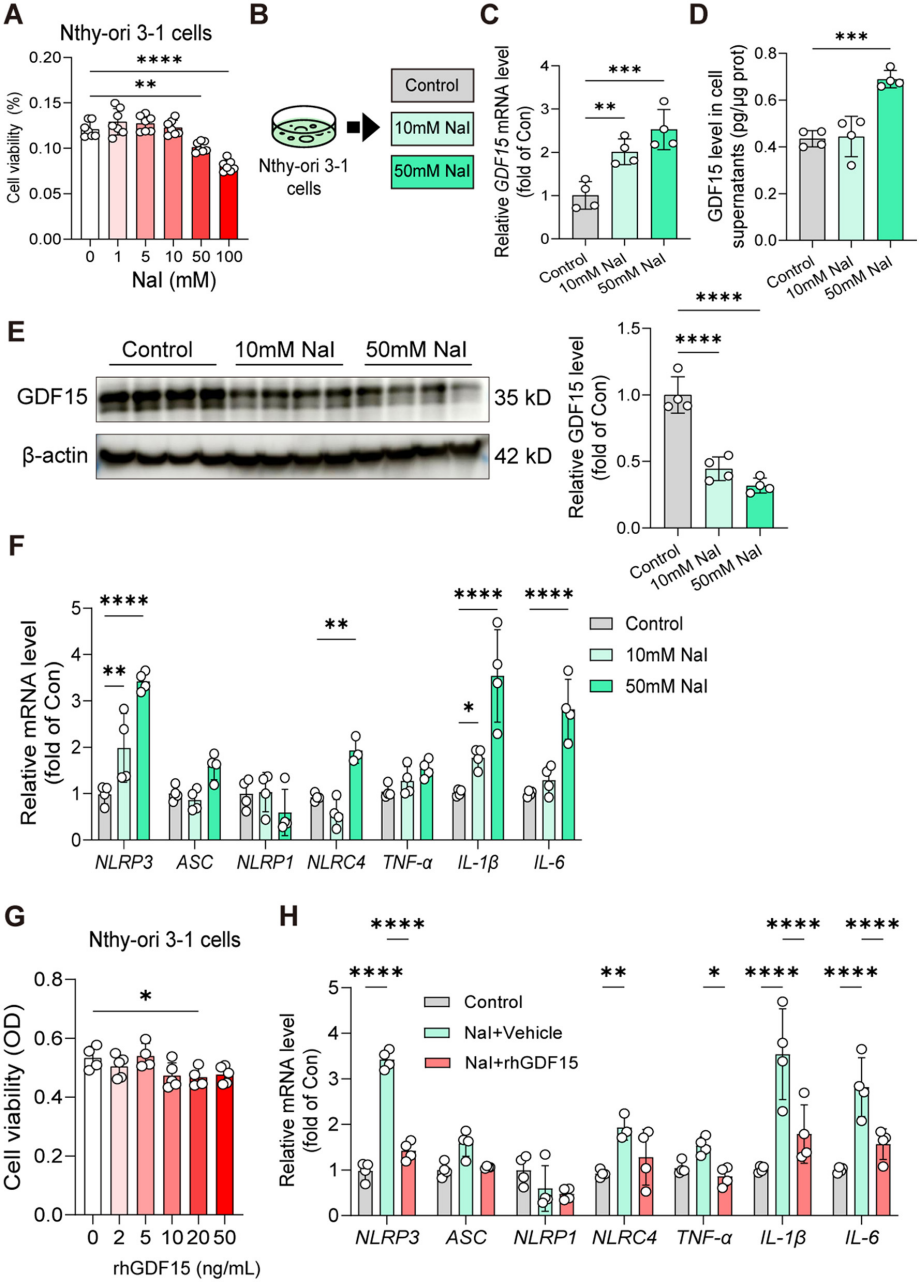
GDF15 attenuates NaI-induced inflammation. **(A)** Cell viability in different concentrations of NaI in Nthy-ori 3-1 cells (*n* = 7). **(B)** The treatment protocol in the NaI-induced Nthy-ori 3-1 cells. **(C)** The mRNA level of *GDF15* in cell supernatants after NaI treatment (*n* = 4). **(D)** GDF15 level in cell supernatants after NaI treatment (*n* = 6). **(E)** Representative gel images of GDF15 after NaI treatment (*n* = 4, left panel) and statistical analysis of protein band gray values (right panel). **(F)** The mRNA level of *NLRP3, ASC, NLRP1, NLRC4, TNF-*α*, IL-1*β and *IL-6* after NaI treatment (*n* = 4). **(G)** Cell viability in different concentrations of rhGDF15 in Nthy-ori 3-1 cells (*n* = 5). **(H)** The mRNA level of *NLRP3, ASC, NLRP1, NLRC4, TNF-*α*, IL-1*β and *IL-6* after rhGDF15 and NaI treatment (*n* = 4). *p < 0.05, **p < 0.01, ***p < 0.001, ****p < 0.0001.

To establish an *in vitro* model of HT, the human normal thyroid follicular epithelial cell line, Nthy-ori 3-1, was treated with varying concentrations of NaI. The cell viability assays (CCK-8) confirmed that 50 mM NaI induced significant cytotoxicity; therefore, 10 mM was selected for subsequent experiments (Figures 5A, B). Following NaI treatment, GDF15 mRNA expression and its concentration in the culture supernatant were significantly elevated, while intracellular protein levels decreased, potentially reflecting enhanced secretion kinetics or translational repression (Figures 5C–E). Furthermore, we assessed the expression of key inflammasome components [NOD-like receptor family pyrin domain-containing 3 (NLRP3), NOD-like receptor family pyrin domain-containing 1 (NLRP1), Apoptosis-associated speck-like protein containing a CARD (ASC), and NOD-like receptor family CARD domain-containing 4 (NLRC4)] and major pro-inflammatory cytokines (TNF-α, IL-1β and IL-6). Notably, NaI treatment significantly upregulated the expression of the NLRP3 inflammasome and the pro-inflammatory cytokine IL-1β (Figure 5F). Based on the above results, high-concentration NaI treatment in Nthy-ori 3-1 cells induces an inflammatory response characterized by NLRP3 inflammasome activation and altered GDF15 expression/secretion, effectively mimicking the pathological environment of HT in vitro.

**Figure 6:**
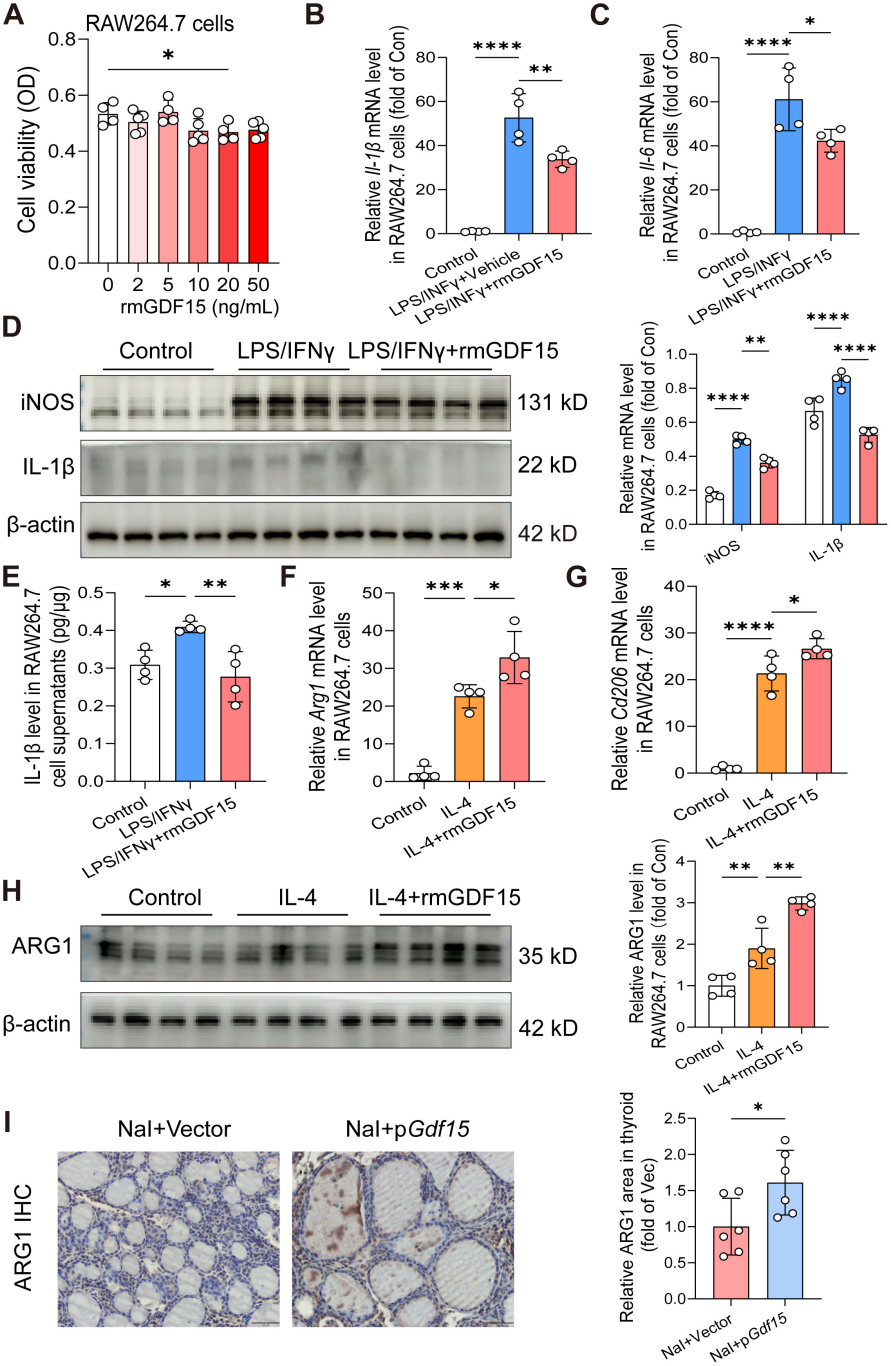
GDF15 promotes M2 macrophage polarization. **(A)** Cell viability in different concentrations of rmGDF15 in RAW264.7 cells (*n* = 5). **(B, C)** The mRNA level of *IL-1*β **(B)** and *IL-6* **(C)** after rhGDF15 and LPS/IFNγ treatment (*n* = 4). **(D)** Representative gel images of iNOS and IL-1β after rhGDF15 and LPS/IFNγ treatment (*n* = 4, left panel) and statistical analysis of protein band gray values (right panel). **(E)** Protein level of IL-1β in cell supernatants after rhGDF15 and LPS/IFNγ treatment (*n* = 4). **(F–G)** The mRNA level of *Arg1* **(G)** and *Cd206* **(H)** after rhGDF15 and IL-4 treatment (*n* = 4). **(H)** Representative gel images of ARG1 after rhGDF15 and LPS/IFNγ treatment (*n* = 4, left panel) and statistical analysis of protein band gray values (right panel). **(I)** Representative images of ARG1 IHC staining (left panel) and statistical analysis of protein band gray values (*n* = 6, right panel) from AAV8-p*Gdf15* and 16 weeks NaI treatment. Scale bar: 50 μm. *p < 0.05, **p < 0.01, ***p < 0.001, ****p < 0.0001.

Previous IHC analyses of human thyroid tissues demonstrated that GDF15 is primarily localized within thyroid follicular epithelial cells (Figure 1G). To elucidate the mechanisms through which thyroid-derived GDF15 regulates concurrent thyroid inflammation and hepatic lipid metabolism in HT, we treated Nthy-ori 3-1 with recombinant human GDF15 (rhGDF15). Based on the results of the CCK-8 cell viability assay, rhGDF15 at a concentration of 10 ng/mL was selected as the optimal dose for subsequent experiments (Figure 5G). Treatment with the rhGDF15 significantly attenuated the NaI-induced upregulation of *NLRP3, IL-1*β and *IL-6* (Figure 5H). Jung et al. (22) demonstrated that GDF15 drives macrophage polarization toward an anti-inflammatory M2 phenotype. Previous F4/80 staining of mouse thyroid sections revealed the anti-inflammatory potential of GDF15. To investigate whether this effect is mediated by the modulation of macrophage polarization, we first determined 10 ng/mL as the optimal concentration of recombinant mouse GDF15 (rmGDF15) using CCK-8 assays (Figure 6A). In macrophages stimulated with LPS and IFNγ, GDF15 treatment significantly suppressed the expression of pro-inflammatory cytokines *Il-1b* and *Il-6* (Figures 6B, C). Similarly, GDF15 decreased the protein level of inducible nitric oxide synthase (iNOS) and IL-1β (Figure 6D). We also measured the level of IL-1β in the cell supernatant, and the results showed that GDF15 significantly inhibited the secretion of the pro-inflammatory factor IL-1β (Figure 6E). In macrophages stimulated with IL-4, GDF15 treatment significantly increased the expression of anti-inflammatory cytokines *Arg1* and *Cd206*, also in protein level of ARG1 (Figures 6F–H). To investigate macrophage polarization, we supplemented our findings with Arg1 expression analysis in thyroid tissues (Figure 6I), which supports the potential for GDF15 to promote an M2-like phenotype.

**Figure 7:**
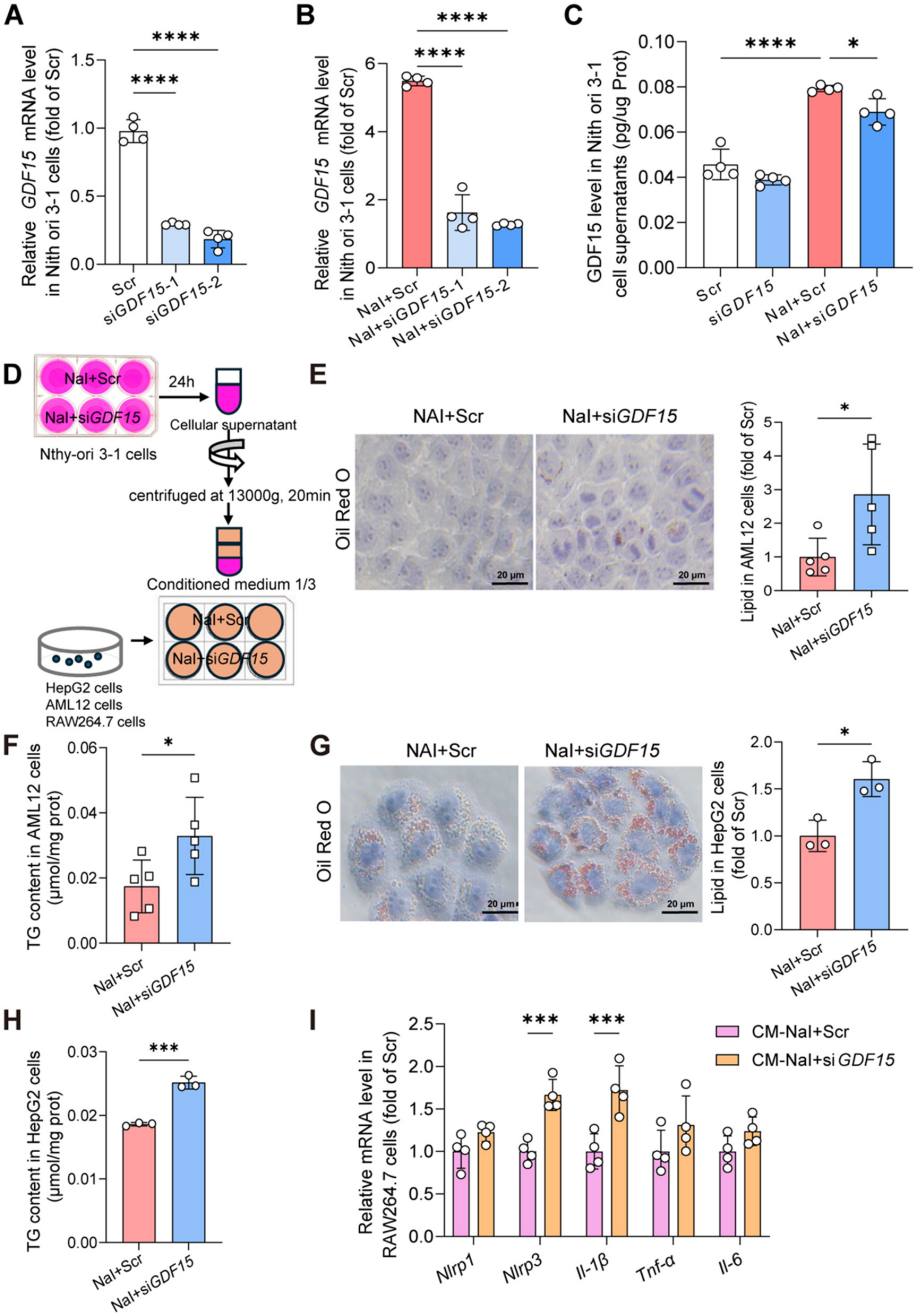
GDF15 knockdown promotes lipid accumulation in hepatocytes and inflammation in RAW264.7 cells. **(A)** The mRNA level of *GDF15* after transfected with si*GDF15*-1 and si*GDF15*-2 in Nthy ori 3-1 cells (*n* = 4). **(B)** The mRNA level of *GDF15* after transfected with si*GDF15*-1 and si*GDF15*-2 and NaI treatment (*n* = 4). **(C)** Protein level of GDF15 in cell supernatants after transfected with si*GDF15*-2 (*n* = 4). **(D)** Conditioned medium treatment protocol. **(E)** Representative images of Oil Red O in AML12 cells (left panel) and statistical analysis of lipid droplet area (right panel). **(F)** TG content in AML12 cells after treated with cell culture supernatant of GDF15 knockdown Nthy ori 3-1 cells (*n* = 5). **(G)** Representative images of Oil Red O in HepG2 cells (left panel) and statistical analysis of lipid droplet area (right panel). **(H)** TG content in HepG2 cells after treated with cell culture supernatant of GDF15 knockdown Nthy ori 3-1 cells (*n* = 4). **(I)** The mRNA level of *Nlrp1, Nlrp3, Il-1*β, *Tnf-*α and *Il-6* after conditional medium treatment (*n* = 4). *p < 0.05, **p < 0.01, ***p < 0.001, ****p < 0.0001.

To further validate the function of thyroid-derived GDF15, we synthesized si*GDF15* and transfected it into cells. The mRNA level of *GDF15* was significantly downregulated, confirming the high knockdown efficiency and successful construction of si*GDF15* (Figure 7A). After transfection into NaI-treated Nthy-ori 3-1 cells, the results showed that si*GDF15* significantly inhibited the NaI-induced upregulation of *GDF15* at the transcriptional level (Figure 7B). Meanwhile, we examined the level of secreted GDF15 in the cell supernatant. The results showed that GDF15 levels in the supernatant were also significantly decreased after transfection with si*GDF15*-2 (Figure 7C). Subsequently, we collected the supernatant to treat HepG2 cells (Figure 7D). The results suggested that GDF15 knockdown significantly aggravated NaI-induced lipid accumulation and increased triglyceride content in AML12 and HepG2 cells (Figures 7E–H). Furthermore, treatment of RAW264.7 cells with the supernatant revealed that GDF15 knockdown promoted the mRNA levels of iNOS and *Il1b* (Figure 7I). Regarding the conditioned medium experiments, we observed that supernatant from siGDF15-transfected thyroid cells aggravated lipid accumulation in AML12 and HepG2 cells and increased NLRP3/IL-1β expression in RAW264.7 cells.

## 4 Discussion

HT, a major autoimmune disease and the leading cause of hypothyroidism, exhibits epidemiological parallels with thyroid tumors, particularly regarding its higher prevalence in females (23, 24). The condition is characterized by an immune-mediated attack by T cells against thyroid tissue, which manifests histologically as lymphoplasmacytic infiltration (25). Our findings revealed increased GDF15 concentrations in the systemic circulation and thyroid microenvironment of HT patients, though the biological significance of this up-regulation in HT remains unclear.

As HT progresses, the risk of hypothyroidism significantly increases. Mild subclinical hypothyroidism can induce dyslipidemia, further elevating the risk of cardiovascular and cerebrovascular complications (26). Analysis of 4,413 participants from the SardiNIA cohort reveals significant correlations between thyroid function and circulating GDF15 concentrations: after covariate adjustment, thyroid-stimulating hormone correlates positively with GDF15 whereas free thyroxine correlates negatively, and individuals positive for anti-thyroperoxidase antibodies exhibit lower GDF15 levels; reduced thyroid hormone may suppress metabolic activity and amplify cellular stress to drive compensatory GDF15 upregulation as a protective response, and further research is needed to clarify the pathophysiological link between anti-thyroperoxidase antibodies and GDF15 (27). Studies show serum GDF15 rises in hyperthyroid patients and falls after antithyroid drug therapy, while thyroid hormones boost GDF15 in mice, proving GDF15 is clinically relevant to human hyperthyroidism (28). Our previous metabolomic analysis identified lipid metabolism as the predominant pathway for differential metabolite enrichment. The concomitant rise in triglycerides in HT patients underscores systemic lipid dysregulation and hepatic accumulation-observations that were further confirmed in a NaI-induced animal model. Some studies have been shown that GDF15 treatment in animal models results in reduced body weight and hepatic lipid accumulation (18, 29–31). GDF15 functions as a stress-responsive protein that is induced by both exercise and metformin (32–34). Systemic GDF15 overexpression was associated with a significant attenuation of NaI-induced TgAb levels and reduced macrophage inflammatory infiltration in thyroid tissue; conversely, GDF15 knockdown correlated with an exacerbation of these pathological changes.

Beyond its metabolic role, it plays a critical anti-inflammatory role in sepsis-mediated organ dysfunction; notably, GDF15 deficiency leads to a exacerbated cytokine storm (35). By orchestrating metabolic control and immune homeostasis, GDF15 has emerged as a promising therapeutic target for metabolic disorders (36). Dai et al. (37) reported that in vitro-induced GDF15-high macrophages appear to constitute a distinct cellular cluster endowed with intrinsic anti-inflammatory properties. Yet, GDF15 expressions are highly context-dependent. For instance, in psoriatic lesions, its expression level is negatively correlated with the severity of inflammation, which is distinctly different from the conventional stress-induced upregulation pattern (38). GDF15 deficiency exacerbated LPS-induced cardiac and renal injury, a process mediated by elevated inflammatory cytokine levels (35). GDF15 interacts with MYPT1 to block AKT-mediated YBX-1 phosphorylation at serine 102, thereby abrogating its nuclear translocation, then Sequestration of YBX-1 in the cytoplasm dampened NLRP3 inflammasome activation and IL-1β secretion, which are potent mediators of inflammation and oxidative stress in SICM (39). Evidence suggests that autoimmune thyroiditis involves extensive macrophage infiltration (40). We observed that F4/80-positive macrophage infiltration was increased in both HT patients and NaI-induced mouse models. Systemic GDF15 overexpression was associated with reduced thyroid inflammation, characterized by limited macrophage infiltration and a shift toward M2 macrophage polarization. Conversely, GDF15 knockdown correlated with more severe inflammatory responses, suggesting a potential link between GDF15 abundance and the regulation of the thyroid immune microenvironment. As a stress-responsive member of the TGF-β superfamily, GDF15 expression is modulated by p53, mitochondrial impairment, and inflammatory signaling (41). Under mitochondrial stress, the integrated stress response (ISR) is triggered, promoting the secretion of GDF15 as a metabokine to mediate distal intercellular communication and metabolic adaptation (42).

Consistent with the characteristics of secretory cytokines, NaI treatment induced robust transcriptional activation of GDF15, resulting in significantly elevated *GDF15* mRNA levels. Upon stress stimulation, newly synthesized GDF15 protein was rapidly exported to the extracellular space via the endoplasmic reticulum-Golgi secretory pathway. This efficient protein secretion rapidly depleted the intracellular GDF15 protein pool, leading to reduced intracellular GDF15 expression detected by Western blot. Concurrently, secreted GDF15 protein accumulated substantially in the culture supernatant. These findings demonstrate that enhanced protein secretion, rather than intracellular proteolysis, is the primary mechanism underlying the apparent discrepancy between mRNA abundance and intracellular protein levels.

Regarding the conditioned medium experiments, we observed that supernatant from si*GDF15*-transfected thyroid cells aggravated lipid accumulation in AML12 and HepG2 cells and increased NLRP3/IL-1β expression in RAW264.7 cells. While these results provide suggestive evidence for a thyroid-liver-macrophage regulatory axis, we acknowledge that the conditioned medium may contain other factors altered by NaI. Future studies using recombinant GDF15 rescue or neutralizing antibodies are required to definitively establish the causal dependency on GDF15.

In summary, our findings demonstrate that elevated GDF15 expression within thyroid follicular epithelial cells from HT patients, and this protein exhibited correlative links with hepatic lipid accumulation. Systemic GDF15 overexpression appeared to correlate with attenuated thyroid inflammatory infiltration and alleviated hepatic lipid deposition, a phenomenon that may be associated with the modulation of M2 macrophage polarization.

### 4.1 Significance, translational potential and limitations

The current study reveals the presence of an immune-metabolic regulatory mechanism in Hashimoto's thyroiditis, providing a potential therapeutic perspective by targeting metabolic pathways. Furthermore, this research provides initial evidence regarding GDF15 expression in thyroid tissue and its potential involvement in the pathogenesis and progression of HT.

Our study had several important limitations. First, the patient cohort requires further expansion to comprehensively characterize the role of GDF15 in thyroid pathophysiology. Second, the anatomical constraints of the murine thyroid present significant technical challenges, including high procedural difficulty for in situ microinjection and substantial animal attrition. Additionally, the use of the Tg promoter often results in expression levels significantly lower than those achieved with the CMV promoter, leading to limited efficiency in achieving robust tissue-specific overexpression or knockdown, compounded by the lengthy duration required for preliminary experiments. Consequently, our study was constrained by a modest sample size, reliance on a high-dose NaI-induced model rather than spontaneous HT models, and the absence of direct in vivo GDF15 intervention. Critically, the lack of thyroid follicular cell-specific GDF15 transgenic or knockout models precludes definitive confirmation that the observed phenotypes originate exclusively from thyroid-derived GDF15. Furthermore, while we identified an association between thyroid-derived GDF15 and hepatic lipid accumulation, establishing a direct causal link requires further mechanistic investigation. Future studies employing hepatocyte-specific GFRAL knockout mice or primary human hepatocytes will be essential to clarify the relationship between thyroid GDF15 and hepatic lipid metabolism.

Collectively, our results demonstrate that GDF15 is stress-induced in the thyroid tissues of patients with HT and in a NaI-induced mouse model, and its expression is associated with thyroid hormone levels. Observations suggest that GDF15 overexpression may be linked to the alleviation of thyroid inflammation and hepatic lipid accumulation, whereas GDF15 knockdown appears to be associated with the exacerbation of these lesions. Given the limitations of the NaI-induced model and the absence of thyroid-specific genetic manipulation, these findings point to a significant association between GDF15 and HT, though further research is required to establish definitive causality.

## Data Availability

The raw data supporting the conclusions of this article will be made available by the authors, without undue reservation.
