# Supplementary material for: Thyroid follicular cell-derived GDF15 attenuates inflammation and lipid dysregulation in Hashimoto's thyroiditis
Source: Front Med (Lausanne). 2026 Jul 22;13:1854594. doi: 10.3389/fmed.2026.1854594 (PMC13439409; doi:10.3389/fmed.2026.1854594)
Supplement: Supplementary file 1 [file Supplementary_File_1.docx]

**Supplemental Figures**

**Fig. 1. AAV8 vector map.**

**Fig. 2. Metabolomic analysis in patients of HT**

A) Schematic overview of the human study. B) Volcano plot of differentially expressed metabolites identified through metabolomic analysis. C) KEGG pathway annotation of differential metabolites.

**Fig. 3. GDF15 overexpression and knockdown ameliorates inflammation and lipid accumulation in HT mice.**

A-B) Fasting body weight (A), liver/body weight ratio (B) were determined in mice 12-16 weeks after NaI treatment (n = 6). C-D) Serum TC (E) and FT3 (D) levels were measured in mice 16 weeks after NaI treatment (n = 6). E-F) Fasting body weight (E), liver/body weight ratio (F) were determined in mice 12-16 weeks after NaI treatment (n = 6). G-H) Serum TC (E) and FT3 (D) levels were measured in mice 16 weeks after NaI treatment (n = 6).
